# Ionizing radiation modulates vascular endothelial growth factor expression through STAT3 signaling pathway in rat neonatal primary astrocyte cultures

**DOI:** 10.1002/brb3.1529

**Published:** 2020-02-27

**Authors:** Guijuan Zhou, Yan Xu, Bing He, Rundong Ma, Yilin Wang, Yunqian Chang, Yangzhi Xie, Lin Wu, Jianghua Huang, Zijian Xiao

**Affiliations:** ^1^ The First Afliated Hospital of University of South China University of South China Hengyang China; ^2^ Leiyang People’s Hospital Leiyang China

**Keywords:** astrocytes, glial fibrillary acidic protein, radiation‐induced brain injury, signal transducer and activator of transcription 3, vascular endothelial growth factor

## Abstract

**Background and Purpose:**

Radiation‐induced brain injury (RBI) usually occurs six months to three years after irradiation, often shows cognitive dysfunction, epilepsy, and other neurological dysfunction. In severe cases, it can cause a wide range of cerebral edema, even herniation. It seriously threatens the survival of patients and their quality of life, and it becomes a key factor in limiting the radiation dose and lowering the therapeutic efficacy in recent years. Therefore, studying the pathogenesis of RBI and exploring new therapeutic targets are of great significance.

**Methods:**

In our study, we observed the activation and secretory function in astrocytes as well as the intracellular signal transducer and activator of transcription 3 (STAT3) signal transduction pathway activation status after exposing different doses of X‐ray irradiation by using MTT, Immunocytologic analysis, and Western blot analysis. Further, we used the same way to explore the role of vascular endothelial growth factor (VEGF) in signal transduction pathways playing in the activation of astrocytes after irradiating through the use of specificInhivascular endothelial growth factorbitors of STAT3.

**Results:**

Ast can be directly activated, reactive hyperplasia and hypertrophy, the expression of the activation marker glial fibrillary acidic protein is increased, and the expression of vascular endothelial growth factor (VEGF) in the cells is increased, which may lead to RBI. After the addition of STAT3 pathway inhibitor, most of the Ast radiation activation was suppressed, and the expression of high‐level expression of VEGF decreased after irradiation.

**Conclusion:**

Our findings demonstrated that X‐ray irradiation directly induced the activation of astrocytes in a persistent manner and X‐ray irradiation activated STAT3 signaling pathway. As the same time, we found that X‐ray irradiation induced the activation of astrocytes and secretion cytokine. The STAT3 signaling pathway may participate in the pathogenesis of radiation‐induced brain injury.

## Graphical abstract

In this work, we found the changes of vascular endothelial growth factor(VEGF) and glial fibrillary acidic protein (GFAP), and the signal transducer and activator of transcription 3(STAT3) signaling pathways involved in astrocytes exposure to X‐rays may be instructive for improving the efficiency of radiation therapy while reducing damage to normal brain tissue.

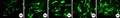

## 1 INTRODUCTION

Radiation‐induced brain injury (RBI) is one of the serious long‐term complications after radiotherapy for head and neck tumor. As many as 200,000 patients receive fractionated whole‐brain irradiation (fWBI) each year (Soffietti, Rudā, & Mutani, 2002; Varlotto et al., 2003), while the incidence of central nervous system injury after radiotherapy has also increased year by year. Taking nasopharyngeal carcinoma as an example, radiation‐induced delay brain injury (RIDBI) is the most serious late‐stage complication after radiotherapy for nasopharyngeal cancer, and the incidence of different reports in recent years has reached 3.6%–8.3% (Tuan et al., 2012). In particular, the progressive cognitive impairment occurs in up to 50% of cancer patients who survive 6 months or longer following partial or fWBI (Johannesen, Lien, Hole, & Lote, 2003; Peiffer, Shi, Olson, & Brunso‐Bechtold, 2010). RBI usually occurs several months or years after radiotherapy, but also delayed to 10 years after the end of radiotherapy (Carole et al., 2009; Dropcho, 2010). Its main clinical manifestations are symptoms and signs of nervous system damage. Severe cases can result in disability and death. It not only causes great economic pressure to society and families, but also causes great pain to patients themselves (Liu et al., 2010). However, the pathogenesis of radiation‐induced brain injury is still not entirely clear and there is lack of effective treatment.

The main pathological manifestation of RBI is cerebral angioedema, which mainly involves the abnormal proliferation of blood vessels in the brain (Lumniczky, Szatmári, & Sáfrány, 2017; Sona & Marian, 2015). Among them, vascular endothelial growth factor (VEGF) plays a key role (Andrews et al., 2017). Nowadays, VEGF is suggested to be the most relevant factor underlying microvascular injury in late delayed RBI, which induces increased microvessel permeability and vasogenic brain edema. Up‐regulation of VEGF has been confirmed in both clinical trials and animal experiments on RBI (Jin, Liang, Chen, Liu, & Zhang, 2014). A variety of cellular components in the brain can express VEGF, mainly astrocytes secretion. Astrocytes are the major glial cells in the central nervous system, and it can participate in neuroimmunomodulation (Bylicky, Mueller, & Day, 2018). The static astrocytes quickly become active and secrete lager amount of cytokines, such as VEGF, after irradiation. To date, Janus kinase–signal transducer and activator of transcription (JAK‐STAT) signaling has been proposed to be crucial in promoting astrogliogenesis (Lee, Tan, Cheah, & Ling, 2016). And of the STAT family, the abnormal activation of STAT3 is closely related to the activation and proliferation of glial cells. According to the literature, STAT3 has a binding site on VEGF promoter, and activation of STAT3 pathway protein can up‐regulate VEGF expression, and activated STAT3 can regulate VEGF expression through this binding site (Niu et al., 2002). Therefore, VEGF is an important regulatory gene in the STAT3 signaling pathway. On this basis, we speculate that ionizing radiation may modulate vascular endothelial growth factor expression through STAT3 signaling pathway in rat neonatal primary astrocyte cultures.

## 2 MATERIAL AND METHODS

### 2.1 Preparation of primary astrocyte cultures

Conventional primary astrocyte cultures were prepared from Sprague‐Dawley rat cerebral cortexas with some modifications. In brief, after removal of the meninges, postnatal day 1 (P1) ratcerebral cortex was minced and digested in 0.125% trypsin (GIBCO, USA) for 10 min in a air bath at 37°C. Enzyme‐digested dissociated cells were triturated with Dulbcco's modified Eagle's medium/nutrient mixture F‐12 ham's (DMEM/F12 1:1; GIBCO, USA) in the presence of 10% fetal bovine serum (GIBCO, USA), washed and centrifuged at 300 × *g* for 5 min. The pellet was resuspended in DMEM/F12 containing 10% fetal bovine serum (FBS), passed through a 10‐μm nylon mesh, washed, and centrifuged at 300 × *g* for 5 min. Following dilution with DMEM/F12 containing 10% FBS and 1% penicillin–streptomycin, the cells were plated on poly‐l‐lysine‐coated culture flasks at the density of 1.0 × 10^6^ cells/ml and allowed to adhere for one hour in a humidified CO2 incubator at 37°C. Next, the adherent fibroblasts were removed, the suspension with nonadherent cells was plated on another poly‐l‐lysine‐coated culture flasks, and fresh DMEM/F12 was added. The cells were adherent to culture flasks after about one day and maintained in DMEM/F12 for nine or ten days with a medium change every two to three days. For passage, primary astrocyte cultures were thoroughly agitated in an orbital incubator shaker at 280 rpm and 37°C for 18 hr on Day 9 or Day 10 after their establishment. Immediately after agitation, all cells suspended in the culture medium were discarded, and attached monolayers were rinsed with phosphate‐buffered saline (PBS) and then dislodged by trypsinization (0.25% trypsin and 0.02% ethylenediaminetetraacetic acid, EDTA) for 5 min at 37°C and plated on poly‐l‐lysine‐coated flasks at the density of 5.0 × 10^4^ cells/ml. Passaged astrocyte cultures between two and three weeks in vitro were used throughout, unless otherwise specified. Purity of astrocyte was confirmed by staining for astrocyte‐specific glialfibrillary acidic protein (GFAP), resulting usually > 99%. All experimental manipulations were approved by the Ethics Committee on Animal Experiment in The First Affiliated Hospital of University of South China and conducted under the control of the Guidelines for Animal Experimentation.

### 2.2 Exposure of cells to ionizing radiation

Cells were cultured in DMEM/F12 + 10% (v/v) fetal bovine serum as above. Before irradiation, treatment of cultured astrocytes was done in the presence or absence of STAT3 inhibitor AG490 (10 μM), which was added 1 hr before irradiation to be used to block STAT3 in this study. Cells treated with equivalent concentration of DMSO were used as controls. Astrocyte cultures were exposed to 20 Gy of X‐ray using a 6 MV X‐ray linear accelerator (Varain 23EX, USA) at dose rate of 400 cGy/min at room temperature. In all experiments, the same experimental design and separate controls of nonirradiated cells were used for all regimens. Zero time is designated as the time point at which exposure to radiation ceased. Cell cultures were harvested for various assays at 4 hr, 12 hr, 24 hr, or 48 hr after irradiation.

### 2.3 Cell viability assay

Cell viability was evaluated by the assay of the conversion of 3(4,5‐dimethylthiazol‐2‐yl)‐2,5‐diphenyltetrazolium bromide (MTT), based on the ability of viable cells to reduce MTT, giving rise to a purple formazan salt. Briefly, primary cultured rat astrocytes were grown in 24‐well plates at a concentration of 1 × 10^5^ cells/ml followed by proper treatment. After incubation for 1 hr at 37°C with 5 mg/ml MTT in Hank's balanced salt solution, the purple reaction product was dissolved in dimethyl sulphoxide. The spectral photometric absorbance of the samples was determined at a wavelength of 540 nm. MTT assay reflects the metabolic activity of cells and serves as a helpful indicator of cell viability. Statistical analyses were conducted by the nonparametric Wilcoxon test.

### 2.4 Immunocytologic analysis

Cultures were fixed for 25 min in cold 4% paraformaldehyde (PFA) in PBS at room temperature. After being washed three times (5 min each time) with PBS, fixed samples were permeabilized in 0.3% Triton X‐100 solution for 20 min and blocked with 5% normal goat serum for 1 hr at room temperature. Primary antibodies and the mouse antiglial fibrillary acidic protein antibody (anti‐GFAP; 1:1,000; Cell Signaling Technology, USA) in the blocking solution were applied overnight at 4°C. After rinsing, samples were incubated with secondary antibody conjugates for mouse IgG (1:200, Cell Signaling Technology) in blocking solution for 2 hr at room temperature. After completion of the secondary antibody incubation, the nuclear stain 4,6‐diamidino‐2‐phenylindole dihydrochloride (DAPI) was added for 5 min. Excess secondary antibody and nuclear counterstain were removed by washing in PBS. Fluorescent‐labeled samples were coverslipped with fluorescent mounting medium. Images were captured using an epifluorescent microscope (Olympus, Japan) equipped with the digital microscope system (Olympus, Japan).

### 2.5 Western blot analysis

Retinal cultures grown on plastic plates were washed with ice‐cold PBS; then, ice‐cold freshly prepared RIPA buffer was added to the well and cells were dislodged using a sterile, disposable cell scraper. Lysates were placed on ice for 30 min, then centrifuged at 16,000 g for 15 min at −4°C, and supernatant fractions were stored at −80°C. Samples were dissolved in 4 × Laemmli sample buffer and boiled for 5 min. The protein concentration was determined using the Micro BCA Protein Assay Kit (Pierce, Rockford, IL, USA). Proteins (30 μg) were separated on 12% SDS‐PAGE and transferred to nitrocellulose membranes at 35 V overnight. The membranes were blocked at room temperature in 3% BSA and incubated overnight at 4°C with the following primary antibodies: mouse monoclonal anti‐SNAP25, rabbit polyclonal antisynaptophysin (homemade), and monoclonal anti‐β‐actin from Calbiochem and Oncogene Research Products (Cambridge, MA, USA). The membranes were washed and incubated with the appropriate peroxidase‐labeled secondary antibody (Bio‐Rad, Hercules, CA, USA) for 1 hr at room temperature. After extensive washes, the immunoreactive bands were detected by chemiluminescence coupled to peroxidase activity (Santa Cruz Biotech) and imaged with a ChemiDoc XRS system (Bio‐Rad Laboratories Inc.). Statistical analyses were conducted by the nonparametric Wilcoxon test.

### 2.6 Statistical analysis

The data are expressed as the mean TSEM and analyzed for statistical significance using analysis of variance (ANOVA), followed by Scheffe's test for multiple comparison. A *p* value *<.05 was considered significant.

## 3 RESULTS

### 3.1 Immunocytochemical identification of cultured astrocytes

**Figure 1:**
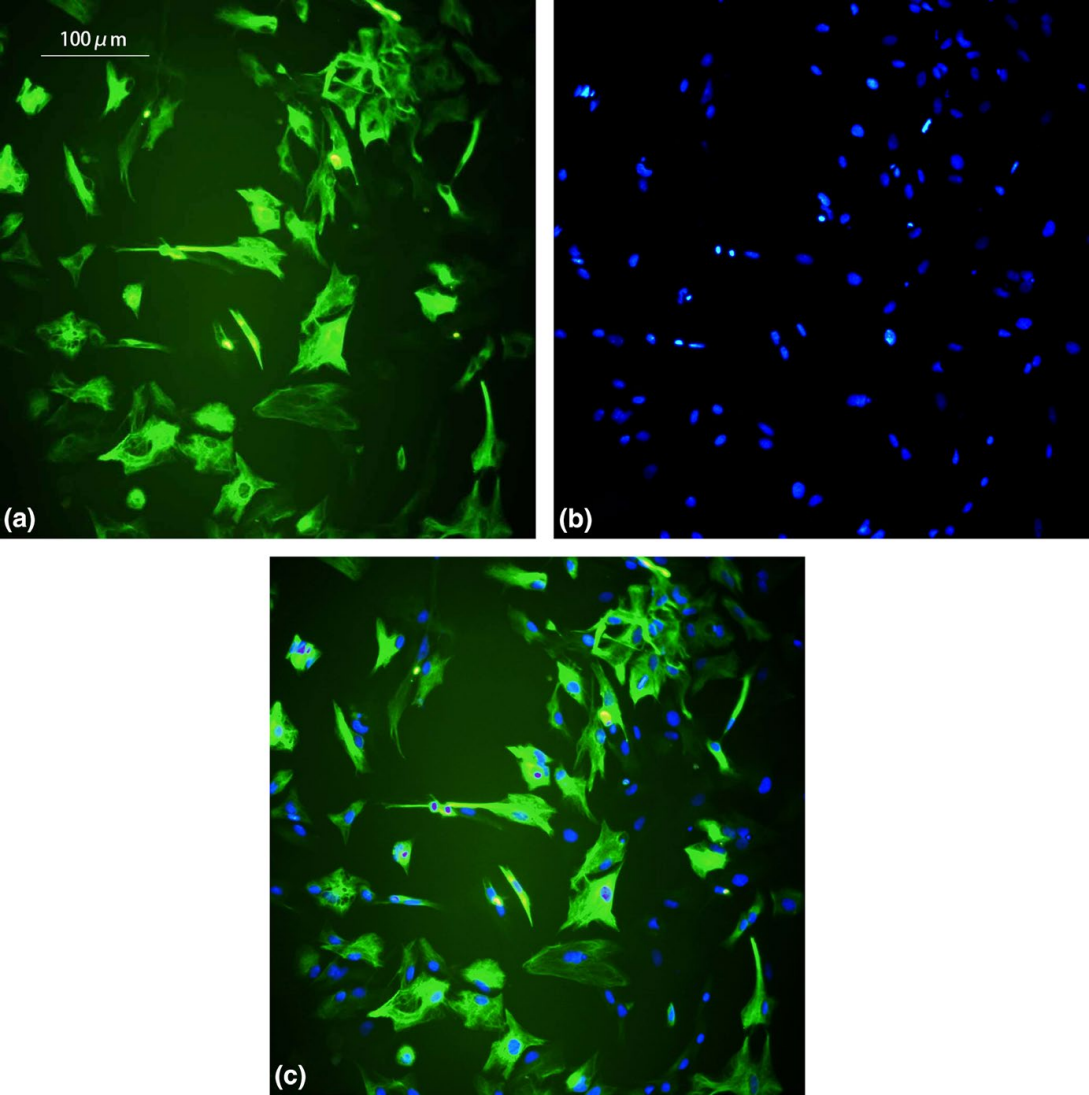
Immunocytochemical identification of astrocytes. Most of them are glial fibrillary acidic protein (GFAP)‐positive cells (×200, a, b, c scale bar = 100 μm). (a) GFAP green fluorescent staining, (b) DAPI dyeing, and (c) Merge

GFAP is an astrocyte‐specific marker, the secondary antibody is FTIC‐labeled green fluorescence, and DAPI is a marker for all nucleus, which is blue fluorescence. After the two images overlap (Merge), astrocytes can be seen the proportion. From Figure 1, it can be seen that most of the cells cultured to the third generation (16 d) are GFAP‐positive cells. The astrocytes are mainly irregular triangles, some of them are polygonal or fusiform, and wining two short protrusion. The majority of the harvested cells were astrocytes (95%).

### 3.2 Astrocyte activation Induced by X‐ray irradiation

#### 3.2.1 Expression changes of astrocyte marker GFAP after X‐ray irradiation

**Figure 2:**
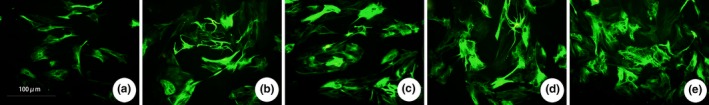
Glial fibrillary acidic protein (GFAP) immunofluorescence staining was used to observe the morphological changes of astrocytes and the expression of GFAP in different dose groups (×320, a, b, c, d, e scale bar = 100 μm). (a) Control (0 Gy), (b) 5 Gy irradiation group, (c) 10 Gy irradiation group, (d) 15 Gy irradiation group, and (e) 20 Gy irradiation group, and all groups were observed at the 48‐hr node

**Figure 3:**
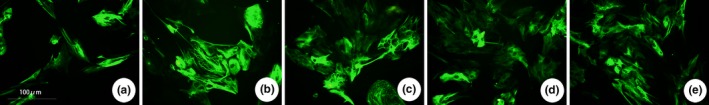
Immunofluorescence staining of glial fibrillary acidic protein (GFAP) to observe the morphological changes of astrocytes and the expression of GFAP at different time points in the 20 Gy dose group (×320, a, b, c, d, e scale bar = 100 μm). (a) Control, (b) 4 hr after 20 Gy irradiation, (c) 12 hr after 20 Gy irradiation, (d) 24 hr after 20 Gy irradiation, and (e) 48 hr after 20 Gy irradiation

**Figure 4:**
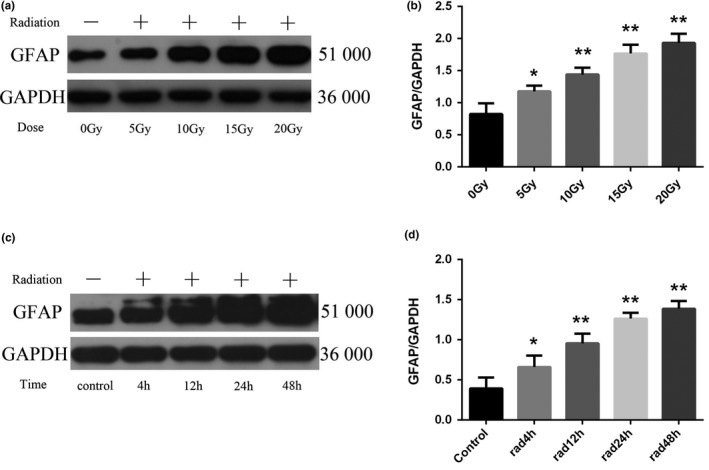
Changes of glial fibrillary acidic protein (GFAP) protein expression in astrocytes of each group (*n* = 5, mean standard deviation). (a, c) GFAP protein expression electrophoresis results. (b, d) Statistical analysis of GFAP protein expression intensity. *Compared with the control group or the 0 Gy irradiation group, *p* < .05. **Compared with the control group or the 0 Gy irradiation group, *p* < .001

GFAP is a cytoskeletal protein of astrocytes, which is recognized as a characteristic marker of astrocytes. Activated astrocytes can cause a significant increase in GFAP, and its expression level can be used to mark the degree of astrocyte damage. From the results of immunofluorescence staining, most of the normal control group had positive GFAP expression, but the number of cells was small, the staining was shallow, the cell body was small, the protrusions were fine, and the number of protrusions was small. After irradiation, the astrocytes in the 5 Gy dose group began to proliferate and degenerate, and the immunofluorescence GFAP staining deepened, the cell body was swollen and swollen, the cell branches increased, and the protrusions thickened. The number of cells positive for GFAP expression was greater than that of the control group *(p* < .05) and the change was more obvious with increasing dose, which can be seen in Figure 2. The 20 Gy dose group began to change in morphology and quantity from 4 hr after irradiation, and as time goes on, the change becomes more obvious, with the mast cells up to 48 hr and GFAP staining in the cytoplasm as shown in Figure 3. The expression of GFAP protein in each group was observed by Western blotting. The results of GFAP protein electrophoresis are shown in Figure 4a and c. The results of statistical analysis of the expression intensity are shown in Figure 4b and d. As can be seen from the figures, compared with the control group, the expression of GFAP protein was increased in the 5, 10, 15, and 20 Gy dose groups, which was consistent with the results of immunocytology. The expression of GFAP increased with the increase of irradiation dose. The expression of GFAP protein began to increase significantly at 4 hr after irradiation in the 20 Gy dose group, and the increase was more obvious with the prolongation of irradiation time. The difference between the groups was statistically significant (*p* < .001).

#### 3.2.2 The viability of astrocytes decreased after X‐ray irradiation

**Figure 5:**
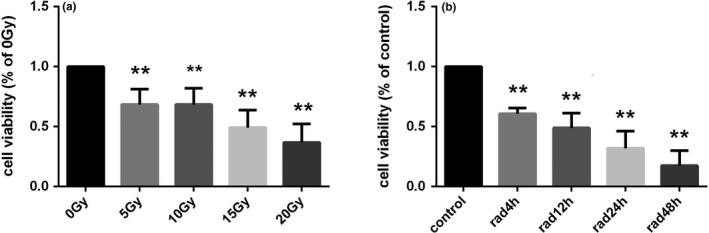
Changes in the viability of astrocytes in astrocytes of each group (*n* = 5, mean standard deviation). (a) viability of astrocytes after X‐ray irradiation. (b) viability of astrocytes at different time points in the 20 Gy dose group. *Compared with the control group or the 0 Gy irradiation group, *p* < .05. **Compared with the control group or the 0 Gy irradiation group, *p* < .001

MTT assay was used to detect the changes of viability of astrocytes after X‐ray irradiation. The results are shown in Figure 5a and b. Compared with the control group, the viability of astrocytes was decreased in the irradiated group (*p* < .001). The intragroup comparison showed that the viability of astrocytes decreased in a dose‐dependent manner with increasing dose. In the 20 Gy dose group, astrocytes viability decreased significantly from 4 to 48 hr after irradiation (*p* < .001).

#### 3.2.3 The vascular endothelial growth factor content increased after X‐ray irradiation

**Figure 6:**
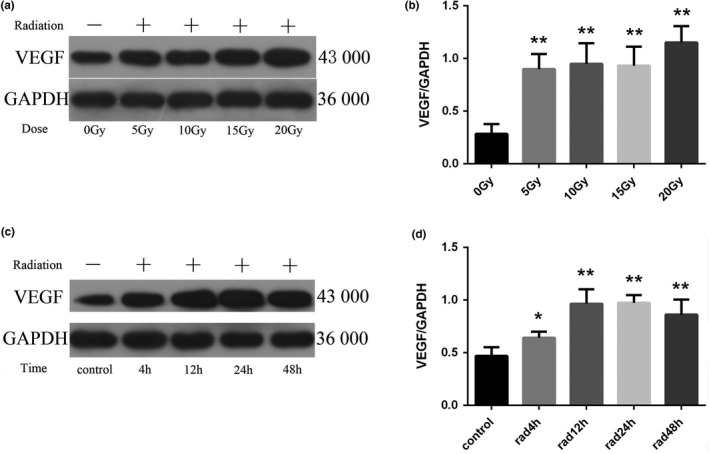
Changes of vascular endothelial growth factor (VEGF) protein expression in astrocytes of each group (*n* = 5, mean standard deviation). (a, c) VEGF protein expression electrophoresis results. (b, d) Statistical analysis of VEGF protein expression intensity. *Compared with the control group or the 0 Gy irradiation group, *p* < .05. **Compared with the control group or the 0 Gy irradiation group, *p* < .001

Western blotting was used to detect the expression of VEGF protein in each group. The results of protein electrophoresis are shown in Figure 6a and c. The results of statistical analysis of the expression intensity are shown in Figure 6b and d. Compared with the control group, the expression of VEGF was increased in the irradiated group (*p* < .001). In the 20 Gy dose group, the expression of VEGF increased significantly from 4 to 48 hr after irradiation (*p* < .05), reached the peak at 24 hr, and decreased slightly after 48 hr, but it was still significantly higher than that of the control group (*p* < .001).

#### 3.2.4 Effect of X‐ray irradiation on phosphorylation of STAT3 in astrocytes

**Figure 7:**
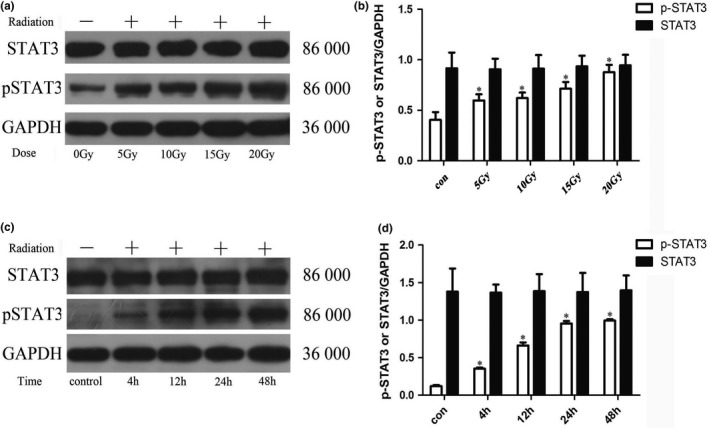
Changes of STAT3 and p‐STAT3 proteins expression in astrocytes of each group (*n* = 5, mean standard deviation). (a, c) STAT3 and p‐STAT3 proteins expression electrophoresis results. (b, d) Statistical analysis of STAT3 and p‐STAT3 proteins expression intensity. *Compared with the control group or the 0 Gy irradiation group, *p* < .05

The results of electrophoresis of STAT3 phosphorylation protein in astrocytes after X‐ray irradiation are shown in Figure 7a and c, and the results of statistical analysis of the expression intensity are shown in Figure 7b and d. As can be seen from the figures, X‐ray irradiation can up‐regulate the phosphorylation level of STAT3 protein in astrocytes. The phosphorylated STAT3 of astrocytes changed significantly after irradiation, and the level of phosphorylation increased significantly, which was higher than that of the control group (*p* < .05), and the phosphorylation level increased with the increase of irradiation dose. At 4 hr after irradiation, the phosphorylation level of STAT3 protein began to increase significantly, and the increase was more obvious with the prolongation of irradiation time, which was dependent on the time after irradiation (*p* < .05). There was no significant change in STAT3 protein changes, and there was no statistical difference.

### 3.3 Effects on astrocyte activation by restraining STAT3 signaling pathway

#### 3.3.1 Changes in GFAP expression after inhibitor pretreatment

**Figure 8:**
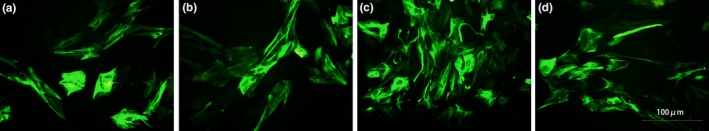
Glial fibrillary acidic protein (GFAP) immunofluorescence staining was used to observe the morphological changes of astrocytes and the expression of GFAP in different dose groups (×320, a, b, c, d scale bar = 100 μm). (a) Solvent control, (b) AG490 control group, (c) Radiation control group, and (d) AG490 + radiation control group

**Figure 9:**
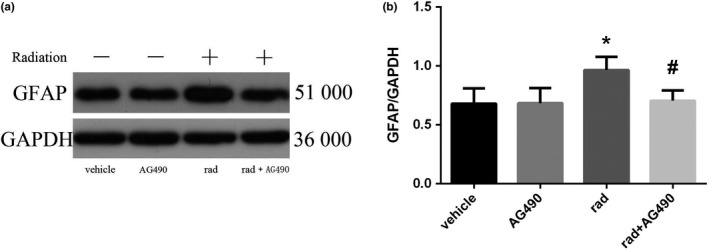
Changes of GFAP protein expression in astrocytes of each group (*n* = 5, mean standard deviation). (a) GFAP protein expression electrophoresis results. (b) Statistical analysis of GFAP protein expression intensity. *Compared with the vehicle group, *p* < .05. ^#^Compared with the rad group, *p* < .05

To verify the activation of astrocytes after inhibitor treatment, we used immunofluorescence techniques to observe changes in the marker GFAP. In Figure 8, the morphology and quantity of the solvent control group and the AG490 alone group were basically the same, the number was small, the staining was shallow, the cell body was small, and the protrusions were fine. Hyperplasia and degeneration occurred after X‐ray irradiation, GFAP staining was deepened, cell body hypertrophy and swelling, cell branching increased, and protrusions were thickened. The morphology and number of cells treated with inhibitor pretreatment were consistent with the solvent control group and the AG490 group alone. The expression of GFAP protein in each group was observed by Western blotting. The results of GFAP protein electrophoresis are shown in Figure 9a. The results of statistical analysis of the expression intensity are shown in Figure 9b. The expression of GFAP protein in the solvent control group and AG490 alone is basically the same, which was no significant differences. The expression of GFAP was significantly increased after X‐ray irradiation, while the group pretreated by inhibitor was significantly lower than that of the simple irradiation group (*p* < .001). There was not significant difference between the control group and the group pretreated by inhibitor.

#### 3.3.2 The viability of astrocytes increased after inhibitor pretreatment

**Figure 10:**
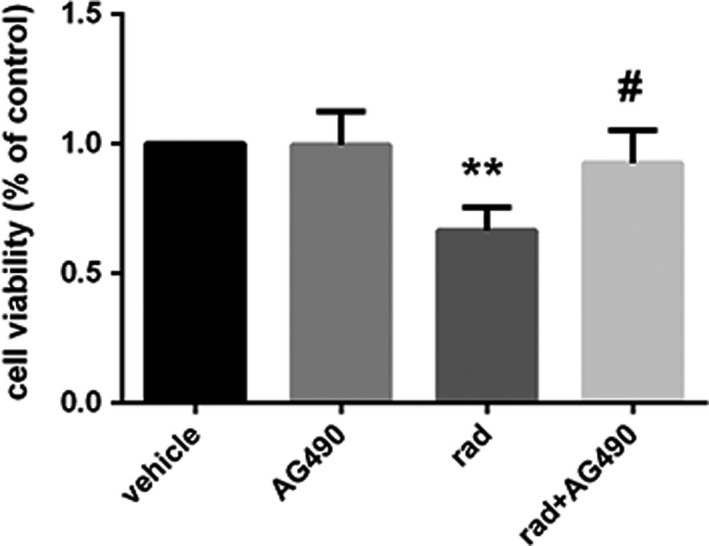
Changes in the viability of astrocytes in astrocytes of each group (*n* = 5, mean standard deviation). **Compared with the vehicle group, *p* < .001. ^#^Compared with the rad group, *p* < .05

The results are shown in Figure 10. The viability of astrocytes between solvent control group and the AG490 group alone was almost the same. Compared with the control group, the viability of astrocytes was significantly decreased in the irradiated group, and there were significant differences (*p* < .001). In the group pretreated by inhibitor, we can see that the viability of astrocytes increased compared to the irradiated group. Compared with the control group, the astrocytes viability was still deficient, while there were no differences.

#### 3.3.3 The expression of VEGF in activated astrocytes declined after inhibitor pretreatment

**Figure 11:**
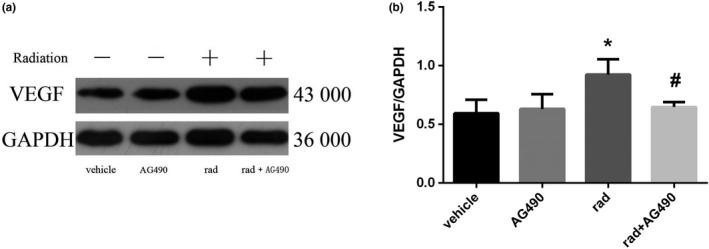
Changes of VEGF protein expression in astrocytes of each group (*n* = 5, mean standard deviation). (a) VEGF protein expression electrophoresis results. (b) Statistical analysis of VEGF protein expression intensity. *Compared with the vehicle group, *p* < .05. ^#^Compared with the rad group, *p* < .05

From Figure 11, we can see that the expression of VEGF in astrocytes was significantly increased in the X‐ray irradiation group compared with the solvent control group and the AG490‐treated alone group. While after pretreatment with AG490, the expression of VEGF significantly decreased, which compared to the irradiated group (*p* < .05).

#### 3.3.4 Changes in STAT3 phosphorylation levels after inhibitor pretreatment

**Figure 12:**
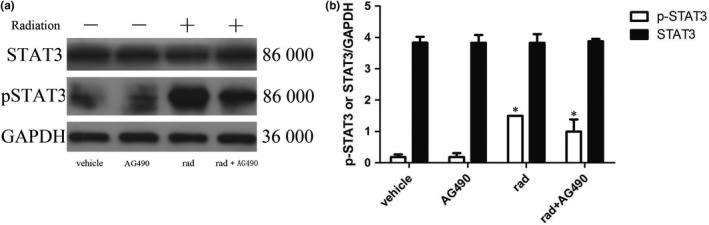
Changes of STAT3 and p‐STAT3 proteins expression in astrocytes of each group (*n* = 5, mean standard deviation). (a) STAT3 and p‐STAT3 proteins expression electrophoresis results. (b) Statistical analysis of STAT3 and p‐STAT3 proteins expression intensity. *Compared with any other group, *p* < .05

From Figure 12, the results showed that the activation of STAT3 in astrocytes was significantly reduced by STAT3 signaling pathway inhibitor AG490 pretreatment compared with the corresponding X‐ray irradiation group, and the phosphorylation level of STAT3 was significantly lower than that of the simple irradiation group (*p* < .05). However, the level of phosphorylation was not significantly lower than that of the control group and the AG490‐treated alone group, and the degree of phosphorylation was still significantly higher than that of the two groups (*p* < .05). It is suggested that AG490 failed to completely block the phosphorylation of STAT3.

## 4 DISCUSSION

The elucidation of the pathogenesis is the basis and premise of clinical intervention, and the pathogenesis of RBI has not yet been determined. Animal experimental research and pathological study of surgical findings found that RBI showed mainly hyperplasia of glial cells, damage of microvascular structure, destruction of blood–brain barrier integrity, apoptosis of nerve cells, abnormal expression of cytokines, etc. (Fauquette, Amourette, Dehouck, & Diserbo, 2012). Traditionally, vascular endothelial cells and oligodendrocytes have been identified as sensitive target cells for ionizing radiation damage. RBI studies at home and abroad have focused on these two types of cells (Xu et al., 2016). For the largest number, astrocytes (Ast), which play an important role in other types of brain damage, are less concerned (Tofilon & Fike, 2000; Yang, Wu, Liang, Rao, & Ju, 2000). As the most common type of cells in the central nervous system, Ast plays an important role in maintaining the homeostasis of the brain microenvironment (Ceyzériat, Abjean, Carrillo‐de Sauvage, Ben Haim, & Escartin, 2016; Zhou, Zuo, & Jiang, 2019). It is closely related to brain tissue damage and postinjury repair and may play a key regulatory role in the pathophysiological process of RBI.

Glial fibrillary acidic protein (GFAP) is a skeletal protein of Ast, which is recognized as a characteristic marker of Ast (Johannesen et al., 2003), how much GFAP is expressed is used to mark the severity of the glial reaction after injury, and can also be used as a marker for Ast activation under pathological conditions (Yang et al., 2000). Under normal conditions, Ast is at rest, with smaller cell bodies, less cytoplasm, shorter protrusions, fewer numbers, and shallower GFAP staining. Ast can be activated rapidly by some external stimuli such as infection, trauma, or physicochemical stimulation. The cell body can become hypertrophy, the protrusion becomes thicker and longer, and the number of protrusions increases. From the results of immunofluorescence staining, most of the normal control group had positive GFAP expression, but the number of cells was small, the staining was shallow, the cell body was small, the protrusions were thin and short, and the number of protrusions was small. After exposure to the irradiation, Ast began to proliferate and degenerate, and the immunofluorescence GFAP staining deepened, the cell body was swollen and swollen, the cell branches increased, and the protrusions thickened. The number of cells positive for GFAP expression was greater than that of the control group. The changes in Ast morphology and GFAP expression were more pronounced with increasing dose. The morphological and quantitative changes occurred in the 20 Gy dose group from 4 hr after irradiation, and the changes were more obvious with the prolongation of time. The mast cells were the most at 48 hr, and the GFAP staining was the deepest in the cytoplasm. We found that the experimental results were consistent with the results of immunocytochemistry. Compared with the 0 Gy group or control group, the expression of GFAP protein was increased in the 5, 10, 15, and 20 Gy dose groups. The expression of GFAP protein began to increase significantly 4 hr after irradiation in the 20 Gy dose group. And the increase was more obvious with the prolongation of irradiation time. It showed continuous activation within 48 hr after irradiation. Therefore, the experimental results clearly indicate that X‐ray irradiation can directly induce the activation of astrocytes.

Vascular endothelial growth factor, known as vascular permeability factor, which is mainly secreted by Ast, can promote the vesicle body activity of endothelial cells and increase vascular permeability by reducing the phosphorylation of tight junction protein between endothelial cells. Overexpression of VEGF is considered the most critical factor in RBI (Zhou, Huang, et al., 2019). Several experimental studies have found that the number of activated Ast increased in the weeks before radiation cerebral edema, the expression of VEGF mRNA in Ast increased, and the level of VEGF protein in Ast around the lesion up‐regulated (Arvold et al., 2005; Wilson, Waleed, Sabek, Zawaski, & Merchant, 2009). Recent foreign clinical trials have confirmed that anti‐VEGF therapy can significantly reduce brain edema in patients with RBI and can continue to relieve the disease (Matuschek, 2011), which suggesting that VEGF may be a key factor in the formation of microvascular damage and radiation brain edema. In our study, Ast was cultured in four different doses of X‐rays, and the expression of VEGF in the cells was significantly increased in a dose‐dependent manner. Similarly, we detected the expression of VEGF in cells at different time after exposure to 20 Gy. We found that the expression of VEGF increased significantly with the prolongation of irradiation time, reached a peak at 24 hr, and decreased slightly at 48 hr, but still significantly higher than the control group. Studies by Nordal RA (Nordal, Andras, Melania, & Shun, 2004) suggest that the expression of VEGF is gradually increased with the prolongation of time after irradiation, which is slightly different from our experimental results. A slight decrease in VEGF expression at 48 hr may be due to the gradual release of VEGF into the cell culture fluid by the secretion of cytokines after increased expression of VEGF in vitro.

Previous studies have shown that the JAK/STAT signal transduction pathways are involved in the development of the central nervous system, such as nerve cell proliferation, survival, and differentiation (Jia et al., 2017). The STAT family is expressed differently in the central nervous system, and signal transduction and transcriptional activator (STAT3) is one of the hallmark proteins of central nervous system injury. STAT3 is mainly expressed in glial cells, and it has been implicated in reactive astrogliosis and plays an important role in regulating cytokine‐mediated signaling pathways (Ceyzériat et al., 2016; Washburn & Neary, 2006). It has been reported that STAT3 has a binding site on VEGF promoter, and activated STAT3 can regulate VEGF expression through this binding site (Niu et al., 2002). Therefore, VEGF is an important regulatory gene in the STAT3 signaling pathway and STAT3 signaling pathway may be an important pathway for ionizing radiation to induce high expression of VEGF. In this experiment, we found that X‐ray irradiation can up‐regulate the phosphorylation level of STAT3 protein in Ast. The phosphorylated STAT3 of Ast showed significant changes after irradiation, and the phosphorylation level gradually increased with the increase of irradiation dose. At 4 hr after irradiation, the phosphorylation level of STAT3 protein began to increase significantly, and the increase was more obvious with the prolongation of irradiation time. There was no significant change in STAT3 protein changes. The experimental results suggested that X‐ray irradiation can activate the STAT3 signaling pathway in astrocytes, and the activation intensity is related to the irradiation dose and the time after irradiation.

In the previous part of the study, we found that X‐ray irradiation can directly induce Ast activation and cell proliferation after activation, and a large number of active products may be involved in the process of nervous system damage. At the same time, it was found that the STAT3 signal transduction pathway, which is an important signaling pathway involved in pathological and physiological processes such as cell proliferation and differentiation and immune inflammation, can be activated by X‐ray. To investigate the role of STAT3 activation in Ast activation, we pretreated Ast with the STAT3 signaling pathway inhibitor AG490 prior to Ast X‐ray irradiation (Zhu, Jia, Luo, & Wang, 2018), and then observed changes in STAT3 phosphorylation and Ast marker GFAP, and related cytokines. The results showed that the expression of VEGF in Ast was significantly increased in the X‐ray irradiation group compared with the solvent control group and the AG490 treatment group alone. However, after AG490 pretreatment, VEGF content decreased significantly compared to the X‐ray irradiation group. It is indicated that AG490 can effectively inhibit the activation and secretion of Ast induced by X‐ray. Compared with the corresponding X‐ray irradiation treatment group, the phosphorylation level of STAT3 in AG490 pretreatment group was significantly lower than that in the simple irradiation group, but it was not completely reduced to the solvent control group and AG490 treatment alone. It is suggested that AG490 may fail to completely block the phosphorylation of STAT3. In recent years, many related studies of the STAT3 signaling pathway have confirmed its important role in regulating cytokine signaling and play a key regulatory role in cell proliferation, differentiation and maturation, cellular immune regulation, and apoptosis (Zhou et al., 2013). In our experimental results, Ast activation and activation product expression were significantly inhibited after inhibition of STAT3 signaling pathway. These results indicated that STAT3 signaling pathway activation is closely related to Ast activation. The intrinsic mechanism may be the direct activation of Ast by X‐ray induction, and the activated Ast expression secretes a large number of cytokines. These factors further activate Ast by activating the STAT3 signaling pathway, and finally maintain the continuous activation of Ast.

In summary, a series of pathophysiological changes can occur after X‐r**a**y irradiation of Ast in vitro. Ast can be directly activated, reactive hyperplasia and hypertrophy, the expression of the activation marker GFAP is increased, and the expression of VEGF in the cells is increased, which may lead to RBI. After the addition of STAT3 pathway inhibitor, most of the Ast radiation activation was suppressed, and the expression of high‐level expression of VEGF decreased after irradiation, indicating that STAT3 signaling pathway is involved in X‐ray‐induced Ast activation process regulation. Understanding the changes in these factors and the signaling pathways involved in Ast exposure to X‐rays may be instructive for improving the efficiency of radiation therapy while reducing damage to normal brain tissue.

## CONFLICT OF INTERESTS

The authors declare no potential conflict of interests.

## Data Availability

The data that support the findings of this study are available from the corresponding author upon reasonable request.
